# Awaab Ishak and the politics of mould in the UK

**DOI:** 10.1016/j.eclinm.2022.101801

**Published:** 2022-12-15

**Authors:** eClinicalMedicine

The death of Awaab Ishak, a 2-year-old who died in December, 2020, as a result of prolonged exposure to mould in his home, sent shockwaves through the UK. The inquest into his death, held on Nov 15, 2022, and subsequent Report to Prevent Future Deaths have prompted serious questions about the social housing system and social determinants of health. As the Coroner leading the inquest pertinently asked, “How in the UK in 2020 does a two-year-old child die as a result of exposure to mould?”

In the UK, social housing is provided by councils in coordination with housing associations (not-for-profit organisations). Housing associations act as the landlords and are responsible for the management and upkeep of homes in line with the Decent Homes Standard—the government’s minimum standard for social housing. When these standards are not met, landlords are obligated to make repairs in a reasonable timeframe. If they do not, tenants can begin a legal disrepair process.

Awaab’s father started such a legal process and repeatedly complained about the mould. Despite this, the housing association managing the property, Rochdale Boroughwide Housing (RBH), made no efforts to treat the mould, improve ventilation, or track the source of the damp. Crucially, the duration of exposure to the toxic mould was a key factor in Awaab’s death. Awaab died of severe granulomatous tracheobronchitis leading to acute airway oedema and cardiac arrest—indicating that spores from the mould entered his respiratory system and, over time and from continued exposure, caused chronic inflammation (as granulomas) that eventually occluded his airway. If the family had been rehoused or repairs made, Awaab might have been saved. A disrepair manager from RBH even inspected the home 6 months before Awaab’s death, noting the extensive mould across three rooms. And still nothing was done*.*

This problem is not confined to RBH or social housing. Substandard housing is a major public health issue. In 2020, 2·2 million homes in England had at least one hazard in the highest risk category and 941 000 had serious damp (English Housing Survey). The scale is estimated to be worse in the private sector, which lacks access to the Housing Ombudsman Service to log complaints. Damp and mould are associated with various adverse health effects, including asthma, respiratory infections and related symptoms, dyspnoea, hypersensitivity pneumonitis, and allergic alveolitis. Other health problems associated with poor housing include infectious diseases, lead poisoning, injuries, and mental ill health. Black residents and people with low income are disproportionately affected.

In light of this public health concern, it is perhaps most worrying of all that the Coroner’s Report to Prevent Future Deaths after Awaab’s inquest points to housing professionals placing too much emphasis on family lifestyle as a potential cause of the mould. RBH have admitted that they made incorrect assumptions when suggesting that the mould could be linked to bathing habits and cooking techniques—a symptom of racial or migrant profiling given that Awaab’s father came to the UK from Sudan in 2016. RBH’s Chief Executive, Gareth Swarbrick, has since been let go and unspecified training has been promised across the whole organisation. But this outcome does not go far enough. Such profiling needs to be consciously avoided by ensuring the focus remains on landlords to provide suitable and safe housing. The Housing Ombudsman Service’s 2021 report on damp and mould recommended just that: landlords should avoid placing the onus on residents and take a “zero-tolerance approach to damp and mould interventions”.

So what can be done? Higher housing standards and proper investment would help. Gas safety and Legionella are tightly regulated, and campaigns to clearly outline landlords’ responsibilities have been beneficial at reducing associated risks. The same information or legislation is not available for damp and mould, so including these hazards in the Decent Homes Standard would be the first step. More proactive inspection is also needed. On investment, chronic underinvestment in housing (social and private) has meant that housing stock is woefully low and poorly maintained, while demand remains high—housing association waiting lists are in excess of 1 million. As a minimum, budgets for building new homes and fixing older homes need to increase, with good insulation prioritised to maintain indoor temperature.

It is well established that cold housing and mould are more dangerous in winter when temperatures fall. Therefore, these problems are likely to get significantly worse against the current backdrop of the energy and cost-of-living crises. 55% of UK households are forecast to fall into fuel poverty by January, 2023, without additional interventions. Households with children, Black and minority ethnic groups, and those on low incomes or living with disabilities are most at risk. More needs to be done to protect these individuals. Short-term fixes, such as warming centres, and short-lived energy price fixes are not sustainable and only go so far. Longer term solutions are needed. The UN’s SDG target 7 of access to affordable, reliable, sustainable, and modern energy by 2030 should serve as a key incentive for the government to implement such solutions.

This problem goes so much further than one house with extensive mould in Rochdale. These widespread failings of substandard housing leading to severe health problems cannot be met with feeble responses of “lessons will be learnt”. The Grenfell Tower fire of 2017 is another devastating example that disproportionately affected individuals from marginalised backgrounds (including many migrants and refugees) and those living with disabilities, after which new laws to protect tenants were promised and are yet to materialise. We hope that this will be a defining moment of reform for the housing sector.

